# Supplementary figures and images for: Weight-dependent susceptibility of tilapia to tilapia lake virus infection
Source: PeerJ. 2021 Jul 6;9:e11738. doi: 10.7717/peerj.11738 (PMC8269736; doi:10.7717/peerj.11738)

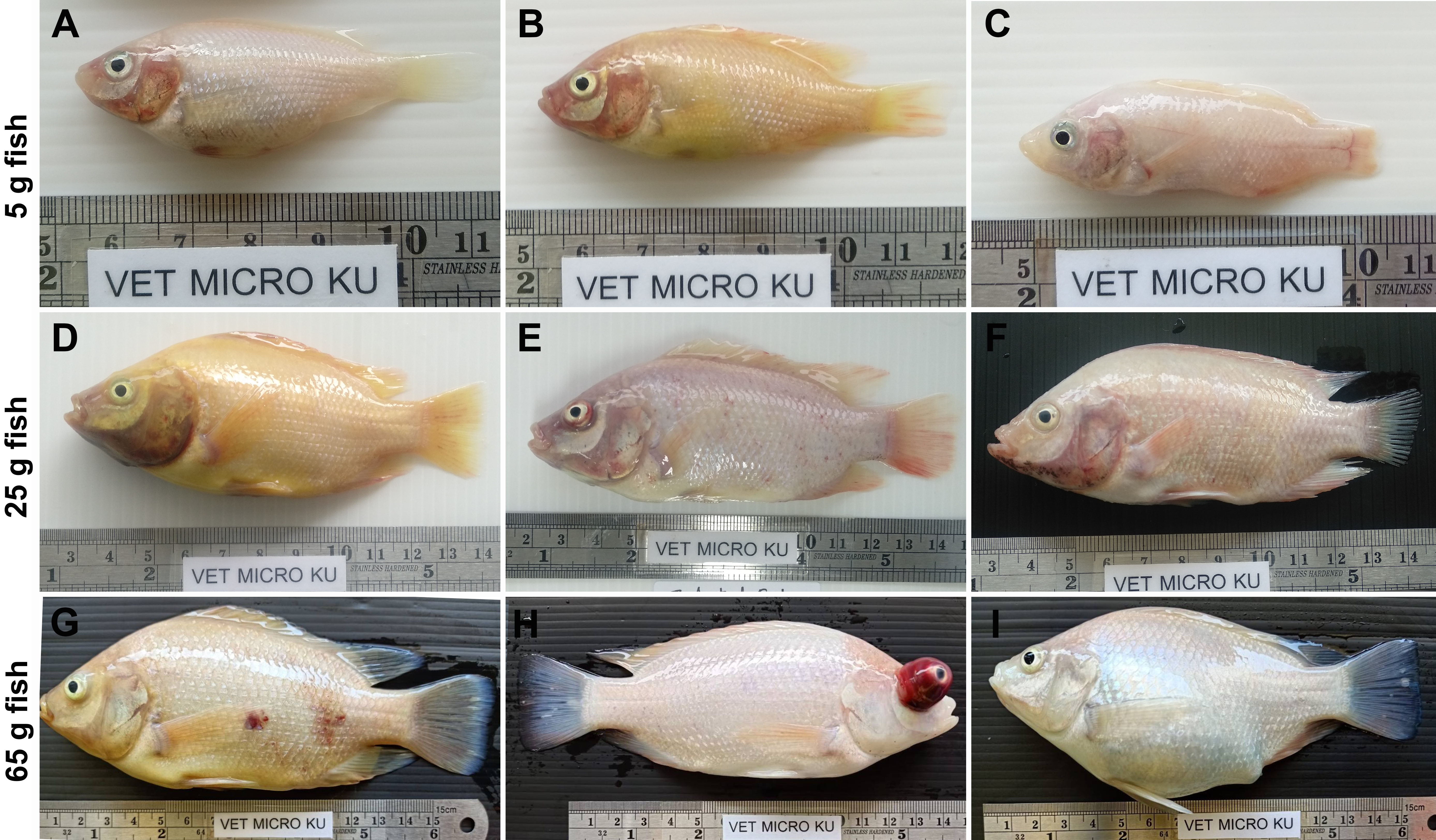

Supplement: Supplemental Information 4 — Representative figures of (A–C) 5 g red hybrid tilapia. (D–F) 25 g red hybrid tilapia. (G–I) 65 g red hybrid tilapia. [file peerj-09-11738-s004.png]

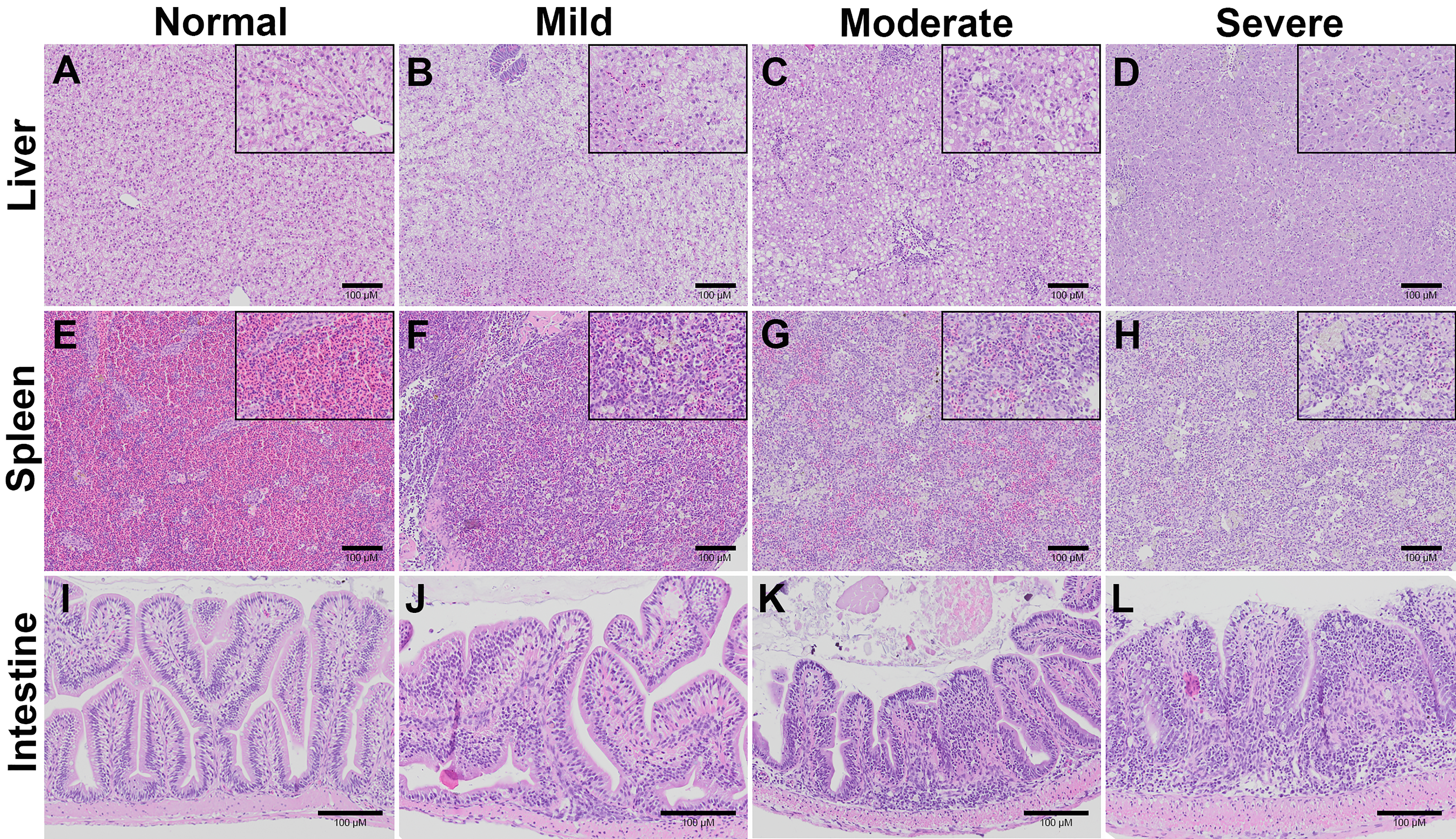

Supplement: Supplemental Information 5 — (A, E, I) Normal liver, spleen and intestine of control fish. (B–D) Liver of TiLV-IP challenge fish with mild, moderate, and severe lesions. (F–H) Spleen of TiLV-IP challenge fish with mild, moderate, and severe lesions. (J–L) Intestine of TiLV-IP challenge fish with mild, moderate, and severe lesions. Lesion scores were graded according to criteria described in Table 1. [file peerj-09-11738-s005.png]
